# Supplementary material for: Contribution of pks+ E. coli mutations to colorectal carcinogenesis
Source: Nat Commun. 2023 Nov 29;14:7827. doi: 10.1038/s41467-023-43329-5 (PMC10687070; doi:10.1038/s41467-023-43329-5)
Supplement: Supplementary file 1 — Supplementary Information [file 41467_2023_43329_MOESM1_ESM.pdf]

Supplementary Figures

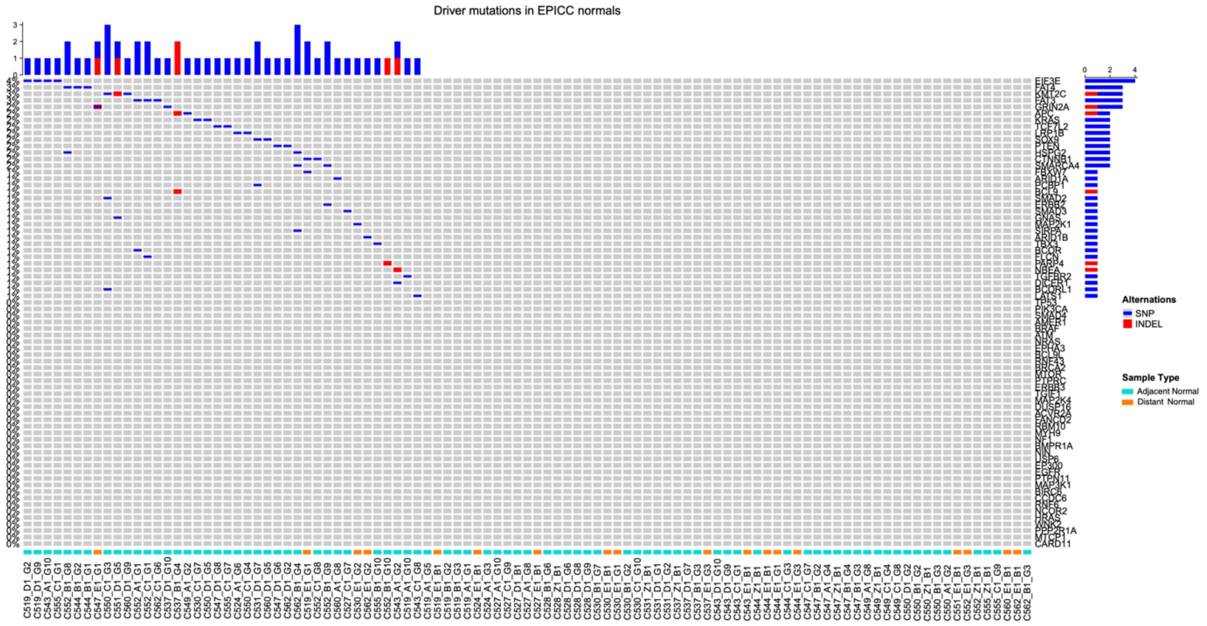

Supplementary Figure 1. *Oncoprint of mutations in colorectal cancer driver genes in normal crypts in our cohort (all samples).*

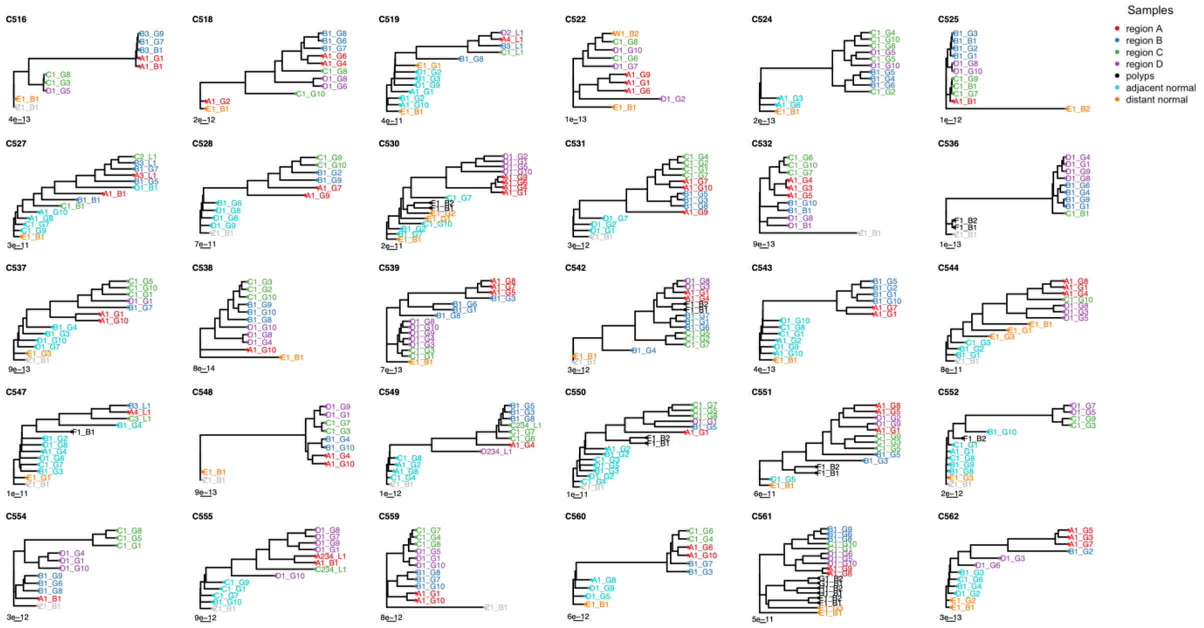

Supplementary Figure 2. Phylogenetic analysis of all crypts (adjacent normal and cancer) from EPICC cohort. The crypts from different regions of the tumor were labelled as ‘A_’,’B_’,’C_’,’D_’, while ‘E_’ are the crypts from distant normal tissues, the cyan coloured labels represent adjacent normals and the black coloured samples are the polyps.

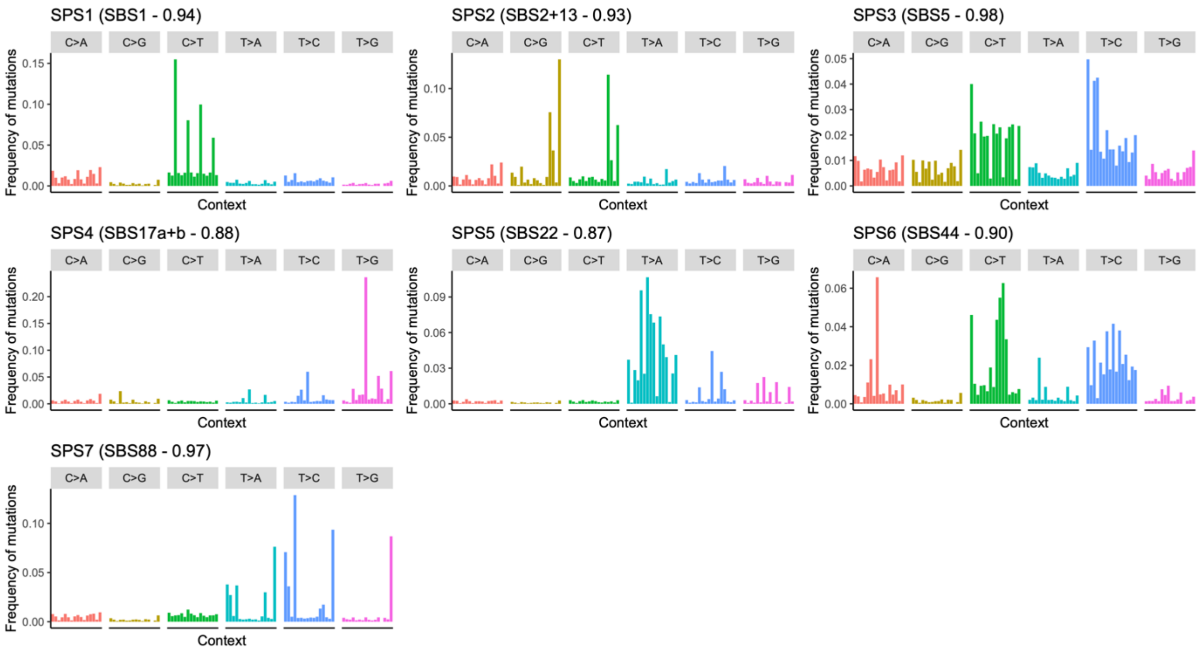

Supplementary Figure 3. The 7 mutational signatures inferred using SparseSignatures. For each signature, we provide the corresponding most similar signature from COSMIC, along with its cosine similarity.

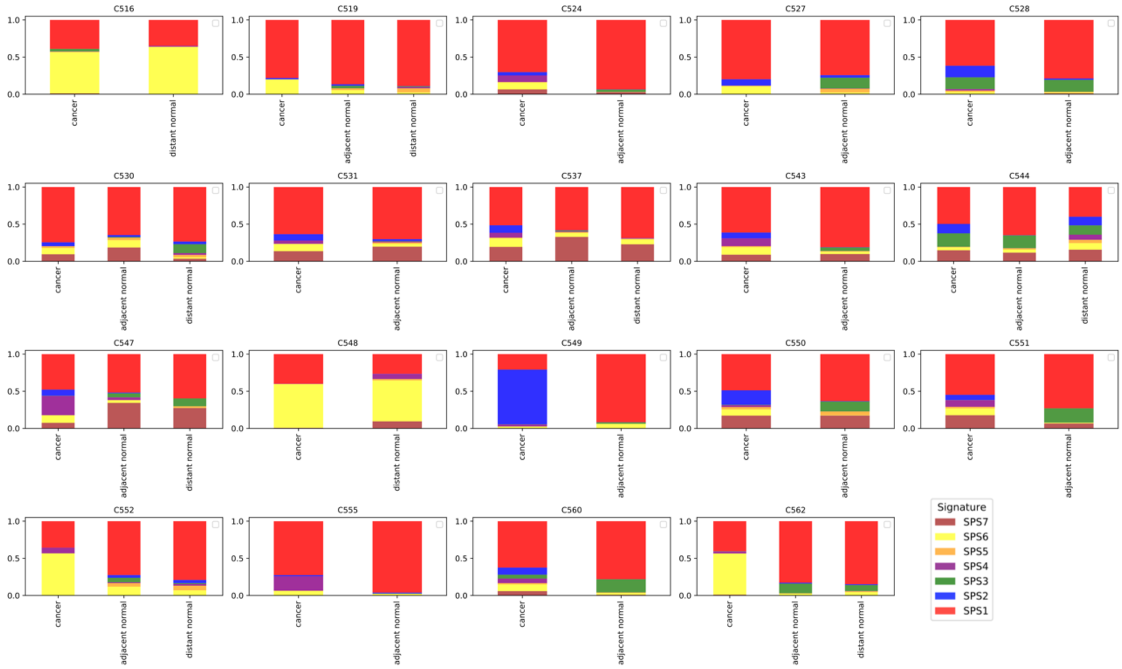

Supplementary Figure 4. Contribution of 7 signatures for the samples from 3 groups(cancer, adjacent normal and distant normal) in EPICC cohort. (Brown coloured bars indicate the SPS-pks)

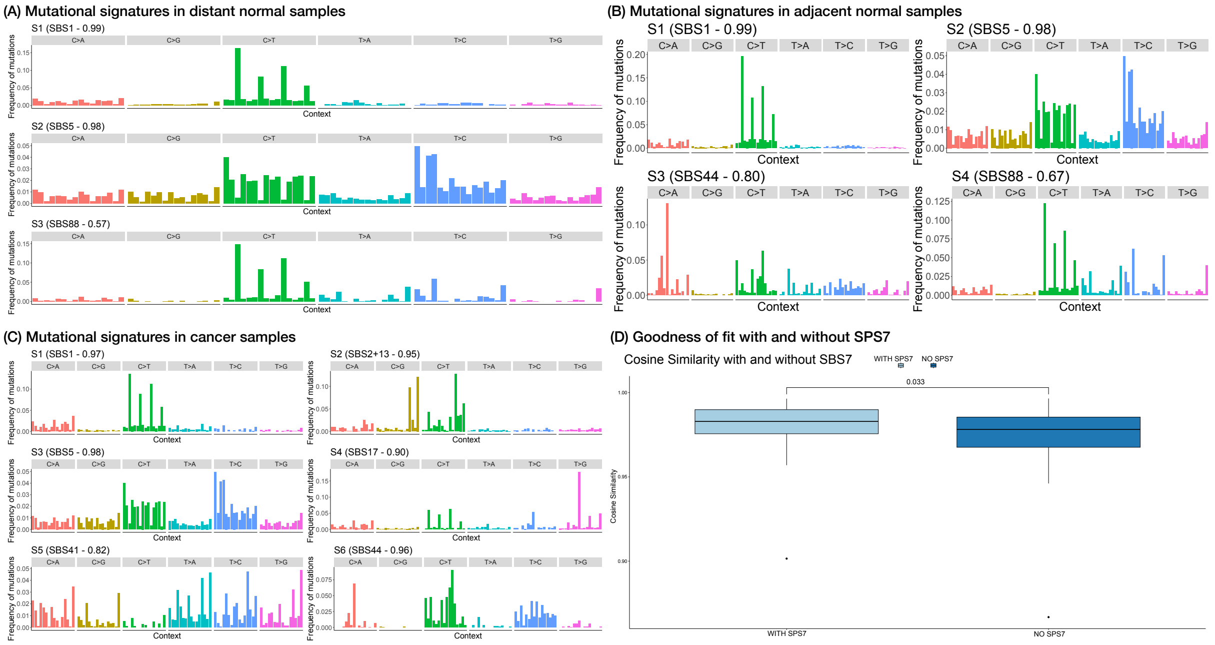

Supplementary Figure 5. (A) Mutational signature extraction for distant normal samples only and **(B)** for adjacent normal samples only. **(C)** Mutational signature extraction for cancer samples only. **(D)** Adding the *pks+* signature to the analysis of the cancer samples significantly improves the fit.

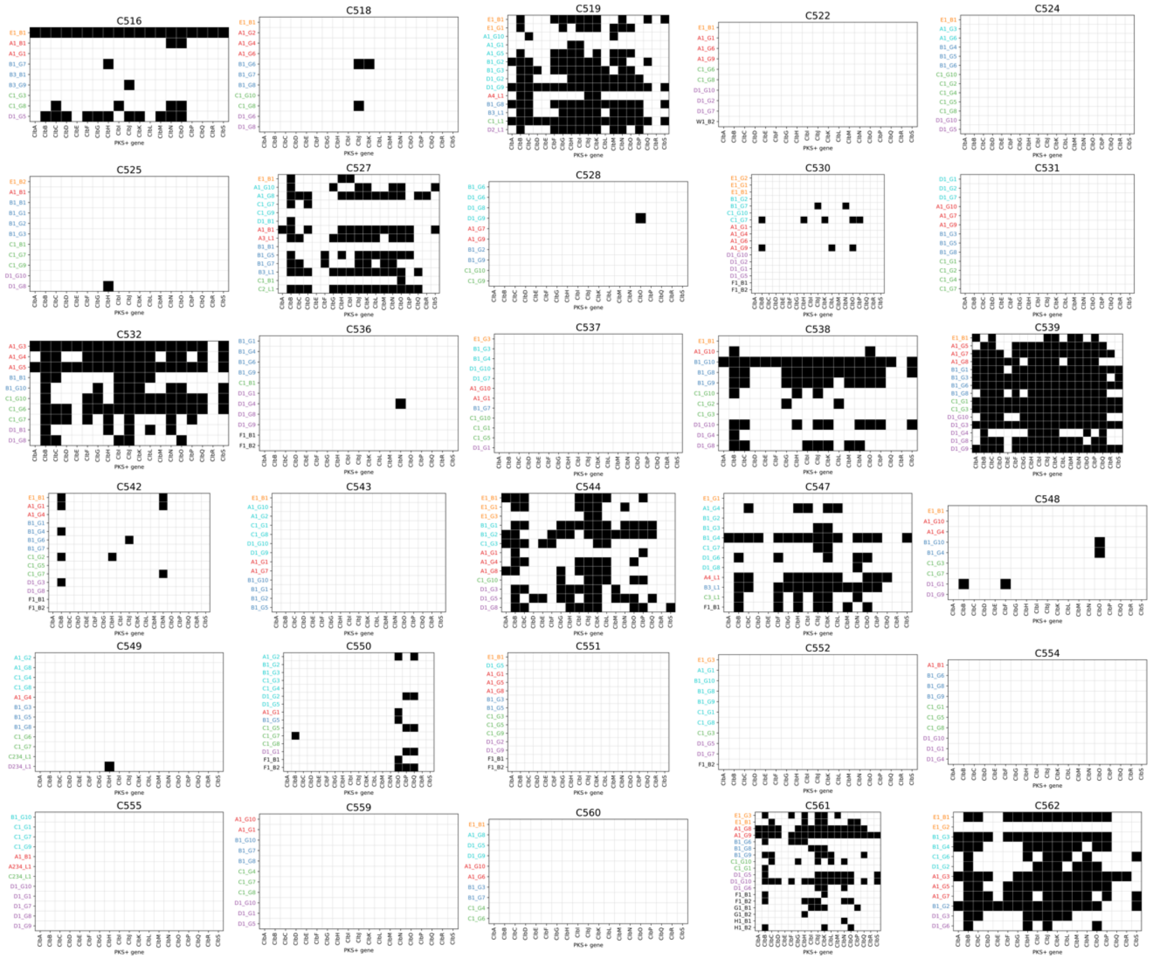

Supplementary Figure 6. Presence of clb genes of all EPICC samples. The black shaded cells indicate the detection of corresponding clb genes in the sample. For each panel, the x-axis represents the clb genes, and the y-axis represents the samples from different regions of the patient.

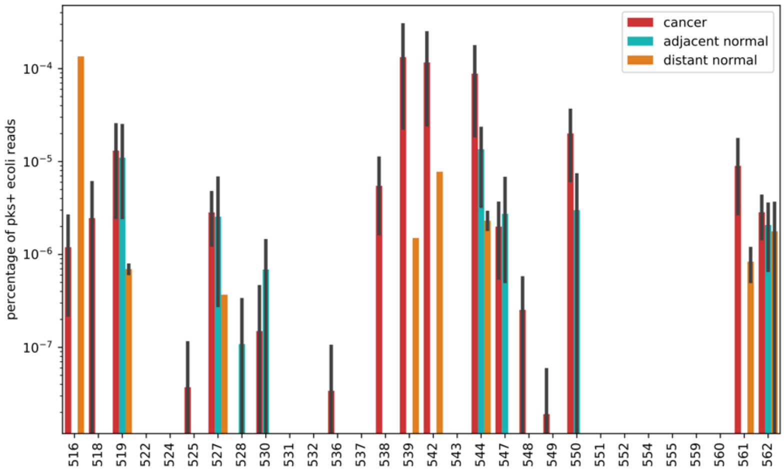

Supplementary Figure 7. Percentage of pks+ e coli reads in samples between 3 groups (cancer, adjacent normal and distant normal samples) from EPICC cohort.

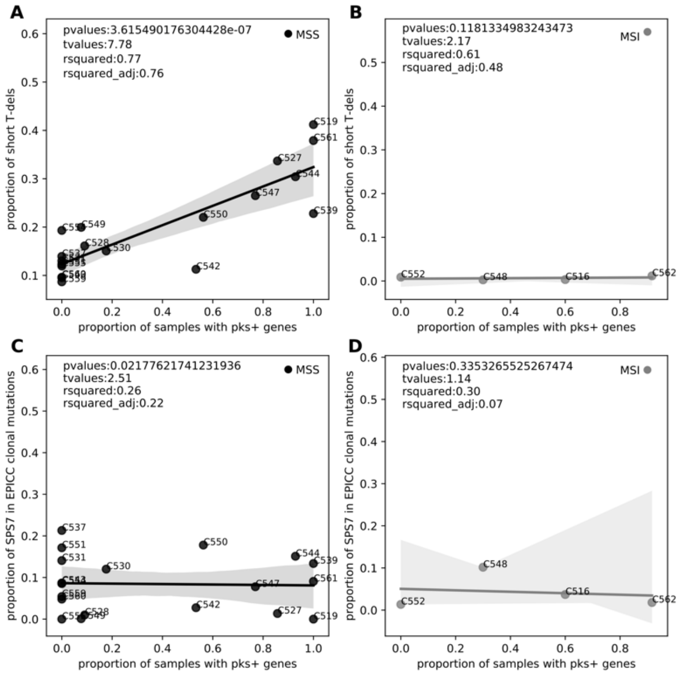

Supplementary Figure 8. (A-B) Correlation between the proportion of samples with pks+ genes and the proportion of short T-homopolymer in MSS and MSI EPICC patients. The X-axis represents the proportion of samples contained reads of clb genes in all samples from

the patients. The Y-axis represents the proportion of single T deletions in short T-homopolymers among all T-homopolymer. **(C-D)** Correlation between the proportion of samples with pks+ genes and the proportion of SPS-pks in clonal mutations of the samples from MSS and MSI EPICC patients.

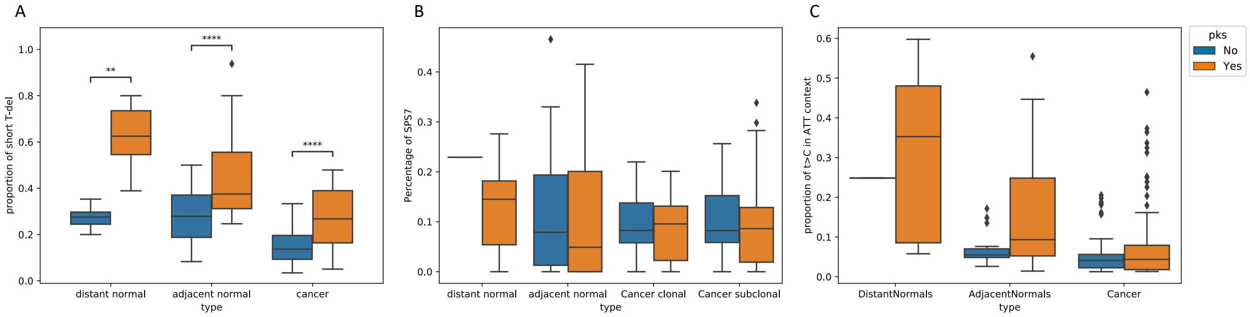

Supplementary Figure 9. (A) Proportion of short T-homopolymer in *pks+* E coli. presence and absence samples from three different groups (distant normal, adjacent normal and cancer). **(B)** Comparison of SPS7 percentage between different groups. **(C)** Proportion of T>C mutations in ATT context for the samples with and without *pks+* E coli. from three groups.

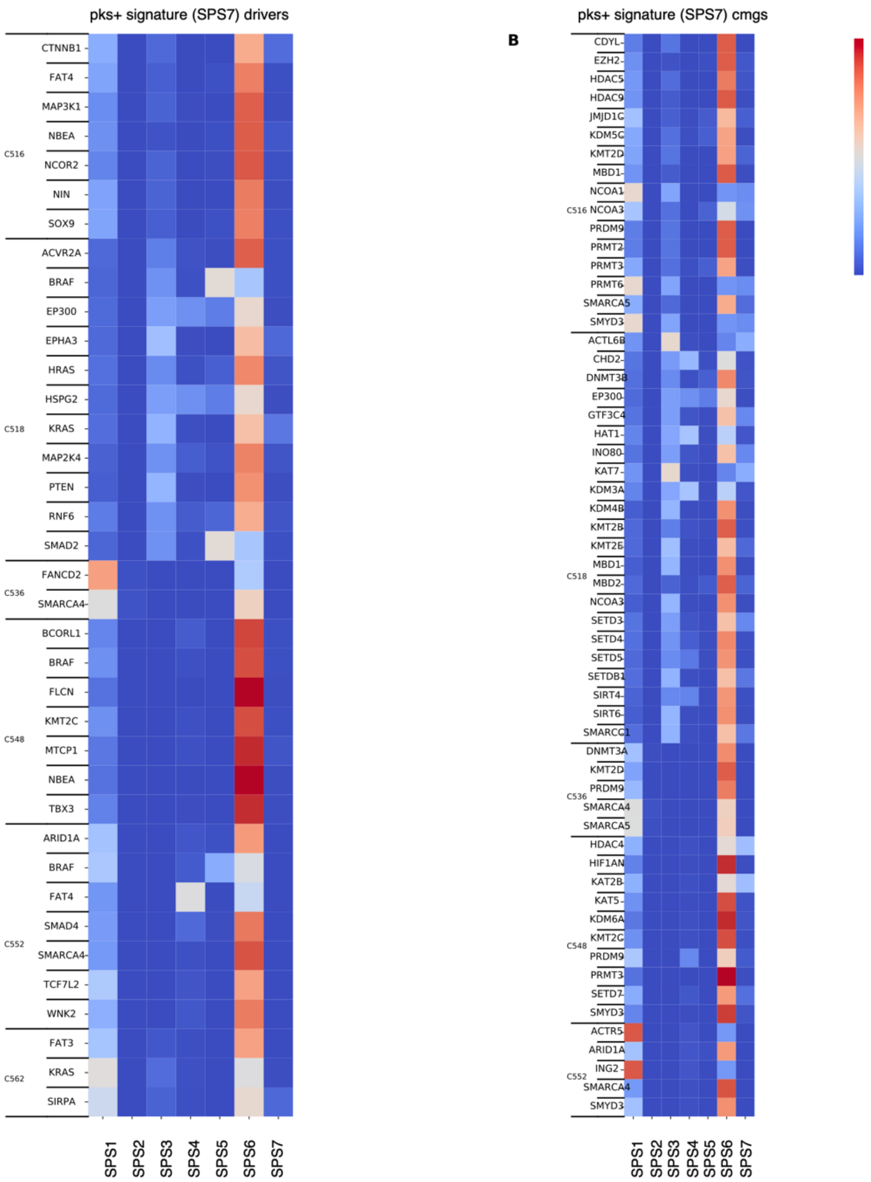

Supplementary Figure 10. Contribution of different mutational signatures to cancer driver and chromatin modifier genes (cmgs) mutations in MSI patients. **(A)** We estimated the probability of SBSs that have caused the non-synonymous mutation on driver genes detected in the patients. **(B)** the SBSs contributions for mutations in chromatin modifier genes.
