# Supplementary material for: Contribution of pks+ E. coli mutations to colorectal carcinogenesis
Source: Nat Commun. 2023 Nov 29;14:7827. doi: 10.1038/s41467-023-43329-5 (PMC10687070; doi:10.1038/s41467-023-43329-5)
Supplement: Supplementary file 3 — Description of Additional Supplementary Files [file 41467_2023_43329_MOESM3_ESM.pdf]

<u>Description of Additional Supplementary Files</u>

Supplementary Data 1: SPSs signatures contribution of all cancer samples(Sheet1), cancer clonal and subclonal signatures(Sheet2) of all EPICC cohort and contribution of Normal samples of Healthy people(Sheet3)
